# *Fit for Dialysis:* a qualitative exploration of the impact of a research-based film for the promotion of exercise in hemodialysis

**DOI:** 10.1186/s12882-018-0984-4

**Published:** 2018-08-06

**Authors:** Pia Kontos, Alisa Grigorovich, Romeo Colobong, Karen-Lee Miller, Gihad E. Nesrallah, Malcolm A. Binns, Shabbir M. H. Alibhai, Trisha Parsons, Sarbjit Vanita Jassal, Alison Thomas, Gary Naglie

**Affiliations:** 1 Toronto Rehabilitation Institute-University Health Network, 550 University Ave, Toronto, ON M5G 2A2 Canada; 2 Dalla Lana School of Public Health, University of Toronto, 155 College St, Toronto, ON M5T 3M7 Canada; 3 Department of Nephrology, Humber River Regional Hospital, 1235 Wilson Ave, Toronto, M3M 0B2 ON Canada; 4 Rotman Research Institute, Baycrest Health Sciences, 3560 Bathurst St, Toronto, ON M6A 2E1 Canada; 5 Department of Medicine, University of Toronto, 1 King’s College Cir, Toronto, ON M5S 1A8 Canada; 6 Institute of Health Policy, Management and Evaluation, 155 College St, Toronto, ON M5T 3M7 Canada; 7 Institute of Medical Sciences, University of Toronto, 1 King’s College Cir, Toronto, ON M5S 1A8 Canada; 8 Department of Medicine, University Health Network, 200 Elizabeth St, Toronto, ON M5G 2C4 Canada; 9 School of Rehabilitation Therapy, Queen’s University, 31 George St, Kingston, ON K7L 3N6 Canada; 10 Division of Nephrology, University Health Network, 200 Elizabeth St, Toronto, ON M5G 2C4 Canada; 11 St. Michael’s Hospital, 30 Bond St, Toronto, ON M5B 1W8 Canada; 12 Lawrence S. Bloomberg Faculty of Nursing, University of Toronto, 155 College St, Toronto, ON M5T 1P8 Canada; 13 Department of Medicine, Baycrest Health Sciences, 3560 Bathurst St, Toronto, ON M6A 2E1 Canada

**Keywords:** Film, Education and training, Exercise/physical activity, Qualitative research methods, End-stage renal disease

## Abstract

**Background:**

Exercise improves functional outcomes and quality of life of older patients with end-stage renal disease undergoing hemodialysis. Yet exercise is not promoted as part of routine care. Health care providers and family carers rarely provide encouragement for patients to exercise, and the majority of older patients remain largely inactive. There is thus the need for a shift in the culture of hemodialysis care towards the promotion of exercise for wellness, including expectations of exercise participation by older patients, and encouragement by health care providers and family carers. Film-based educational initiatives hold promise to effect cultures of best practice, but have yet to be utilized in this population.

**Methods:**

We developed a research-based film, *Fit for Dialysis,* to promote exercise for wellness in hemodialysis care. Using a qualitative approach, we evaluated the effects that resulted from engagement with this film (e.g. knowledge/attitudes regarding the importance of exercise-based principles of wellness) as well as the generative mechanisms of these effects (e.g. realism, aesthetics). We also explored the factors related to patients, family carers, and health care providers that influenced engagement with the film, and the successful uptake of the key messages of *Fit for Dialysis*. We conducted qualitative interviews with 10 patients, 10 health care providers, and 10 family carers. Data were analyzed using thematic analysis.

**Results:**

The film was perceived to be effective in increasing patients’, family carers’ and health care providers’ understanding of the importance of exercise and its benefits, motivating patients to exercise, and in increasing encouragement by family carers and health care providers of patient exercise. Realism (e.g. character identification) and aesthetic qualities of the film (e.g. dialogue) were identified as central generative mechanisms.

**Conclusions:**

*Fit for Dialysis* is well-positioned to optimize the health and wellbeing of older adults undergoing hemodialysis.

**Trial registration:**

NCT02754271 (ClinicalTrials.gov), retroactively registered on April 21, 2016.

## Background

Older adults with end-stage renal disease (ESRD), the largest segment of the patient population undergoing hemodialysis, have lower physical function, which increases the risk for frailty, mobility disability, and cardiovascular mortality [1, 2]. Exercise is recommended for patients undergoing hemodialysis as it has been demonstrated to improve functional capacity [3–5], cardiovascular outcomes [6–8], prevent falls [9, 10], and enhance health-related quality of life [6, 11].

Despite the proven benefits of exercise, and practice guidelines that recommend regular exercise for patients living with ESRD undergoing hemodialysis [12, 13], exercise is not systematically incorporated into routine care, particularly amongst older patients [14–16]. Even when exercise programs are offered as part of hemodialysis care, participation and follow-up exercise adherence rates remain poor [14–16]. Qualitative research exploring the gap between best practice guidelines and nephrology care has identified the need for a shift in the culture of hemodialysis care from the exclusive focus on medical treatment in which exercise is only prescribed for acute conditions (e.g. knee replacement, myocardial infarction), towards a wellness perspective that includes exercise prescription, counseling, and assessment as part of routine care for all medically stable patients [14, 15, 17]. Key to achieving this shift is providing health care providers, patients, and family carers with education regarding the importance of exercise for older adults with ESRD undergoing hemodialysis, and addressing fears of potential exercise-related adverse events. Health care providers and family carers also need education regarding how best to provide verbal and non-verbal encouragement of exercise to patients [15, 17, 18].

Broad-reaching and innovative approaches to education regarding exercise in hemodialysis care are needed. There has been an exponential growth in the use of film for educational purposes, including the influence of perceptions and behaviours regarding health [19–21]. Films of diverse types (e.g. documentary, drama, animation) are used across many domains of health care, including psychotherapy, cancer screening and treatment, diabetes management, and HIV/AIDS risk-reduction [19, 22]. Research suggests that film is an engaging and familiar medium that transcends literacy levels and has broad reach and accessibility [19, 22]. Research-based film can be particularly well-suited to convey health-related information as it captivates the imagination, supports interactive learning, and facilitates critical reflection and emotional engagement [22–24]. Given these strengths, film holds particular promise to effect cultures of best practice.

However, despite the popularity of the use of film for health education, there is a paucity of research exploring its effectiveness, and there has been a call for the development of a stronger evidence base to support its future use [20, 22]. Qualitative research in particular has been identified as helpful for providing an understanding of the factors that influence perceptions of the effectiveness of film-based educational intiatives [19]. Ours is the first study to evaluate perceptions of the effectiveness of research-based film to increase the knowledge/attitudes regarding exercise for wellness, and for motivating patient exercise in the context of hemodialysis care and everyday life for older adults living with ESRD.

### Fit for Dialysis

*Fit for Dialysis* [25] was created to bridge research on exercise with hemodialysis care. The design and development of the film was informed by the theoretical knowledge translation framework, Critical Realism and the Arts Research Utilization Model (CRARUM) [26]. CRARUM is premised on critical realism, which provides a sophisticated understanding of the interrelationship between contextual-, individual-, and intervention-level factors, and how this interrelationship can facilitate or impede the uptake of key messages of the intervention. Further, it remedies the limitations of many knowledge translation initiatives by identifying specific components necessary for the successful implementation of best evidence, and by recommending arts-based methodologies for such initiatives. *Fit for Dialysis* is theoretically-informed [27–31] and aligned with the practice setting which shapes or maintains the targeted attitudes and behaviours [28, 29, 32–34]. Specifically, *Fit for Dialysis* was tailored to the contextual contingencies of out-patient hemodialysis settings in terms of providing “a roadmap to overcome the disciplinary silos and paternalism towards patients which inhibit intradialytic exercise, and addressing some of the life circumstances that may influence patients’ self-management of exercise behaviors at home and in the community” [35] (pg.6). The film was also designed to facilitate critical reflection among health care providers and family carers about the manner in which contextual/cultural factors and processes influence and shape hemodialysis care (e.g. fear of adverse outcomes, family carer protectiveness). Such reflection is deemed particularly essential for practice change initiatives [36–44] to assist health care providers to see how their own practice styles signal underlying assumptions regarding the importance of exercise for wellness vis-à-vis point of care priorities and decisions, and judgments regarding patient safety [16].

*Fit for Dialysis* is a 15-min research-based dramatic film based on an ethnographic study of a hemodialysis unit to explore the culture of hemodialysis care with a particular focus on practices related to exercise participation and support for older adults living with ESRD [15, 35]. Focus groups were conducted with patients, family carers, and health care providers working in nephrology, and naturalistic observations of health care provider-patient interactions during hemodialysis sessions were conducted to explore the factors that enable and constrain patient exercise, and counselling and support regarding exercise. Key themes identified in this research included: patients’ desire for exercise; barriers to exercise such as nurses’ preferences for patient sedentariness during dialysis sessions and family carers’ protectiveness. These themes were used to inform the development of the key messages of *Fit for Dialysis,* which included the importance of exercise for wellness (i.e. intradialytic exercise and exercise in the context of everyday life), as well as the contextual factors that influence participation in exercise and its support. The aim of the film was to convey these messages, to facilitate critical reflection about them, and to foster corresponding change. The process of developing the film was dialogic and interactive; staged readings of the script were conducted with key hemodialysis stakeholders (e.g. patients, family carers, health care providers) and their feedback was incorporated into subsequent iterations of the script.

*Fit for Dialysis* follows the experiences of Marvin, an older hemodialysis patient, and his wife Claire, who are frustrated with Marvin’s deconditioning and weakness because of how it limits their participation in valued activities. Marvin inquires with the health care providers in the hospital where he receives treatment about the possibility of doing exercise during his hemodialysis sessions, but encounters resistance to this idea by everyone he approaches (e.g. the physiotherapist explains that she cannot work with Marvin until he receives an exercise prescription from his doctor; the nurse explains that supporting intradialytic exercise is beyond her professional knowledge). Despite this, Claire strongly encourages Marvin to continue pressing his health care providers to provide him the prescription, opportunity, and equipment to exercise. Marvin persists, and ultimately the physiotherapist collaborates with the nephrologist and nephrology nurses to implement exercise for Marvin. By the end of the film, the individual, social, and familial benefits of wellness through exercise are made clear. In the final scene, Marvin walks down his front steps to pick up the newspaper, surprised by the ease with which he does so given his struggle with this activity prior to engaging in exercise; he turns around to smile at Claire who stands in the doorway equally pleased and smiles back.

## Methods

We conducted an evaluation of the impact of *Fit for Dialysis* on patients, family carers, and health care providers. In contrast to a strictly outcome-based approach that assesses impact using quantitative measures, the approach taken here to evaluate the impact of this arts-based knowledge translation initiative was qualitative and more nuanced and expansive [26, 45]. Specifically, the focus was not only the perceived effects that resulted from engagement with the film (e.g. knowledge/attitudes regarding the importance of exercise-based principles of wellness), but also the process of engagement with the film to explain these effects (e.g. realism, aesthetics). We also explored the factors related to patients, family carers, and health care providers (e.g. nephrologists, nurses, and clinical managers) that influenced their engagement with the film and the successful uptake of the key messages of *Fit for Dialysis*. This qualitative exploration was part of a larger mixed methods study [16]. Here we focus only on the qualitative data collected on a hemodialysis unit in an acute care hospital in urban central Canada.

Patients were eligible to participate if they: (a) had conversational ability in English; (b) were a registered patient in the hospital hemodialysis unit for at least 3 months; (c) received ≥2 in-centre hemodialysis sessions per week; (d) were ≥ 65 years of age; and (e) had a family carer who agreed to participate in the study. The patients’ family carers were eligible if they: a) had conversational ability in English; and (b) provided supervision/assistance with activities of daily living and/or emotional support without financial compensation. All health care providers working at the hemodialysis unit were eligible to participate (e.g. nephrologists, nurses, clinical managers); they received information about the study through an in-service held by author Colobong who had no relationship with the hospital, hemodialysis unit, or individuals associated with it. The nephrologists, in consultation with the Nurse Coordinator of the hemodialysis unit identified eligible study participants. The Nurse Coordinator provided a standardized introduction to the study for patients and family carers. Once approached by the Nurse Coordinator, if they expressed interest in participating in the study, and agreed to the release of their telephone number to author Colobong, they were contacted to schedule a meeting to receive further explanation of the study. After listening to the study explanation, and having any questions about the study answered, all potential participants were given the consent form to review and sign. Of the 88 patients on the unit, 23 patient/family carer dyads met our eligibility criteria, of which 10 dyads (10 patients and 10 family carers) agreed to participate, and 13 dyads declined. Patients were between the ages of 65–89, of which the majority (80%) were men, and family carers were between the ages of 42–73, of which the majority (80%) were women. Of the 43 eligible staff (37 nurses, 5 nephrologists, 1 clinical manager), 10 agreed to participate; ages ranged between 31 and 53 and the majority (80%) were women. A total of 30 individuals participated (see Table 1). Ethics approval was obtained from the research ethics boards of the Toronto Rehabilitation Institute-University Health Network and the institution from where data were collected. All participants provided written informed consent to participate.

**Table 1:** Participant demographics

Stakeholder	Characteristic	Frequency/ Mean ± SD (range)
Patients (*n* = 10)	Women	2
	Men	8
	Age (years)	73 ± 7.9 (65–89)
	Time in dialysis unit (dialysis vintage)	6 < 5 years
		4 ≥ 5 years
Family Carers (*n* = 10)	Women	8
	Men	2
	Age (years)	63 ± 9.9 (42–72)
	Relationship	5 Spouse
		3 Child
		2 other
Health Care Providers (n = 10)	Women	8
	Men	2
	Age (years)	44 ± 6.4 (31–53)
	Profession	4 Nephrologist
		5 Dialysis Nurse
		1 Clinical Manager
	Years worked in Nephrology	12.0 ± 6.6 (1.25–20)

Prior to viewing the film, semi-structured interviews were conducted with all participants to explore their perceptions/experiences regarding: (1) exercise; (2) exercise behaviour; and (3) the barriers and facilitators regarding exercise for patients undergoing hemodialysis (see Table 2 for examples of questions). In order to explore participants’ engagement with the film, their uptake of its key messages, and how such uptake was sustained by them over time, at 8 and 16 weeks after viewing the film, interviews were conducted with all participants. The purpose of the interviews was to explore perceptions of the effectiveness of *Fit for Dialysis* as an educational modality for educating about, and motivating or supporting intradialytic exercise and exercise in the context of everyday life (Table 2).

**Table 2:** Examples of interview questions

Interview time point	Examples of questions
Patients	
Baseline	• What are your thoughts about engaging in exercise during dialysis? • What are your thoughts about exercising on non-dialysis days? • What do you think are the factors that make it possible to exercise during dialysis? What about on non-dialysis days at home and in the community? • What do you think are the factors that hinder exercise during dialysis? • What about on non-dialysis days at home and in the community?
8 and 16 Weeks	• Were there particular scenes that had an impact on you? • What messages did you take away from the film? • Did the film teach you anything you didn’t already know about exercise? • Has the film influenced your involvement in exercise/activity-related behaviours?
Family Carers	
Baseline	• What are your thoughts about the person you care for engaging in exercise during dialysis? • What are your thoughts about him/her engaging in exercise on non-dialysis days at home and in the community? • What do you think are the factors that make it possible for patients to exercise during dialysis? What about on non-dialysis days at home and in the community? • What do you think are the factors that hinder exercise during dialysis? What about on non-dialysis days at home and in the community?
8 and 16 Weeks	• Were there particular scenes that had an impact on you? • What messages did you take away from the film?
	• Has the film influenced how you think about the things that help make it possible for the patient to exercise? • In what ways have you been involved in the exercise of the patient during dialysis? What about on non-dialysis days at home and in the community?
Health Care Providers	
Baseline	• What are your thoughts about your patients engaging in exercise during dialysis? What about patients engaging in exercise on non-dialysis days at home and in the community? • What do you think are the factors that make it possible for patients to exercise during dialysis? What about on non-dialysis days at home and in the community? • What do you think are the factors that hinder exercise during dialysis? What about on non-dialysis days at home and in the community?
8 and 16 Weeks	• Were there particular scenes that had an impact on you? • What messages did you take away from the film? • Has the film influenced how you think about the things that help make it possible for the patient to exercise? • Has the film influenced your involvement in exercise/activity-related behaviours?

All interviews were conducted by author Colobong on the hemodialysis unit or by phone and lasted approximately 60 min. They were all audiorecorded and professionally transcribed, and the transcripts were anonymized. We analyzed the data using a thematic analysis approach beginning with an inductive descriptive process of sorting and defining the data [46, 47]. All interview transcripts were read by the first three authors, descriptively coded, and then text segments were grouped into broad topic-oriented categories related to knowledge/attitudes and preferences and practices related to exercise, and barriers and facilitators regarding exercise participation and support. For example, “I didn’t think that it would be possible for me to [participate in]…exercise” was coded as ‘knowledge gained’, which was later grouped under the theme ‘from disbelief and fear to seeing the benefits of exercise’. Text segments belonging to the same category were then compared within and across all participant interviews. The three authors then discussed these codes and subsequent theme development with the other authors and resolved any discrepancies in interpretation. NVivo 10 was used to facilitate coding. To ensure methodological rigour and trustworthiness of the analysis, we used the following strategies: multiple authors participated in data analysis; a dependability audit [48] was kept which included audio recordings, transcripts, interview guides, and data analysis processes and products (e.g. coding scheme and supporting quotes from the interview transcripts, minutes from analysis meetings, NVivo node reports); frequent reviews were made of the audit by the research team; and sufficient detail was provided in the audit to allow outsider assessment of theoretical generalizability (i.e. ‘fittingness’ of the findings with other contexts) [48].

## Results

We have organized our findings into the following three themes: ‘from disbelief and fear to seeing the benefits of exercise’; ‘motivation and encouragement’; and ‘realism and aesthetics.’ The first theme captures perceptions of the effectiveness of *Fit for Dialysis* in fostering awareness that participation in exercise is possible for older adults with ESRD undergoing hemodialysis and that it has important benefits. The second theme focuses on how the film was perceived as effective in motivating patients to engage in exercise, and family carers and health care providers in encouraging patient exercise. These two themes illustrate how participants experienced changes in their understanding of the importance of exercise and in their participation in exercise or encouragement of exercise. The consistency in participants’ reported uptake of key messages from the film between 8 and 16 weeks suggests that the changes they experienced were sustained over time. The final theme refers to the importance of the realism of the film and its aesthetic qualities (e.g. characters, story line, dialogue) for engagement with the film and supporting its perceived effectiveness.

### From disbelief and fear to seeing the benefits of exercise

Across the three groups of participants (health care providers, patients, and family carers) there was enhanced awareness that participation in exercise is possible and can be safe for patients undergoing hemodialysis, and that it has important benefits. For example, when asked what were the key messages that they took from the film, participants from each group described how it helped them to see that contrary to the dominant assumption that patients with ESRD undergoing hemodialysis are too medically unstable to exercise, exercise was in fact both possible and beneficial for these patients:

[B]efore I thought that when you’re on dialysis … I didn’t think that it would be possible for me to [participate in] … exercise. (Patient).

Well, the big one was that you could actually be active during the dialysis. That was an education for me, and an education for my dad too … You know, it was a real eye-opener for us…like wow, you can do that? (Family carer)

The film importantly allayed fears that exercise could have an adverse effect on patients. This was expressed by both family carers and health care providers alike:

I learned that it’s not so scary to do exercise, that it’s a good thing … [and] to let [patients] see that they’re … not so limited [in what they can do] (Family carer).

[M]ost of our patients are afraid of exercising and the family sometimes tries to protect them and they don’t allow them to do much … the spouses, you know, are very protective and they don’t let them even … go for shopping or for walks, because they think that if they are on dialysis something horrible is going to happen … [and even] we didn’t know what … to say to the patients about exercise and how to advise them. And we were afraid, I think. So now I don’t think I’m so afraid of [exercise]. … Well [the film] showed that, you know, if exercise is done … supervised and with some rules in place, so that patients don’t over-exert themselves, they do quite well and they can do more and more every day. (Nephrologist).

[Before the film] I had no idea [what] exercise [was] going to look like … I imagined [patients] on the stationary bikes [and] I was worried … I was negative. Because the way I imagined this look [sic] like, I didn’t want … anything bad happening. So that’s why when you don’t know, you don’t know what to expect and of course you have negative thoughts. Thinking, “Oh my gosh, you’re looking after that patient. His blood pressure will go higher, will drop. He’ll drop from the bike and what’s going to happen?” I literally told them [this could happen] – now I’m laughing [about this]. (Nurse)

In addition to the film allaying fears, it was effective in highlighting the benefits of engaging in exercise. For example, a patient expressed that the film “showed me the benefit. It showed me what you could achieve after doing [exercise]. And that’s what it taught me”. Similarly, a nephrologist noted that after seeing the film:

I think that now we can start … telling [patients] that in the first place that exercise is not forbidden for them, right? They can start doing exercises, and they can do them in the unit or at home, and that keeping active is important for them and for the[ir] quality of life, and it comes with many other benefits, from, you know, cardiovascular point of view. Bones, everything else.

### Motivation and encouragement

The film was effective not only in changing attitudes and perceptions regarding exercise in hemodialysis care, but also was described by patients as motivating them in exercise, and described by family carers and health care providers as motivating them to encourage patient exercise. For example, in many cases patients’ motivation to exercise was attributed to seeing the benefits of exercise for Marvin, the protagonist in the film:

It just motivated me. It motivated me and showed me the benefits of it from the beginning of the movie to the end. [In] the beginning, [Marvin] wasn’t motivated, he wasn’t sure, he felt ill. And then at the end his whole outlook changed, his whole demeanour changed not just physically. And that’s what I wanted to do, I wanted my demeanour to change, I wanted to feel energized. You know … I’m sick and tired of being sick and tired. I wanted to feel that energy that that gentleman showed that he had after his exercise … and that he felt, even not just physically, but mentally. Changed his whole outlook. And that’s what … I wanted. (Patient).

I said [to myself] if I exercise [I] may become same [sic] like Marvin, doing better walking. Better. Marvin did start walk better off. [Seeing Marvin] I feel like I could do the same thing like Marvin, or even better. (Patient).

Family carers and health care providers described how the film prompted them to encourage patient exercise. For family carers, in some cases, the encouragement was attributed to seeing the importance of encouragement for sustaining patient exercise in the film. For example, in response to a question about the key take-away messages from the film, a family carer responded: “the person doing dialysis can do exercises, it’s good for them, and the family can support them.” Similarly, a family carer reflected on how the film influenced her involvement in her father’s exercise:

Only to be encouraging to him, that was it … [W]atching the film … I can envision him sitting in the chair doing this exercise … because I saw what he would be able to do. I was able to relate and say [to him] “oh yeah, well that’s great … I like what you’re doing and it’s great.”

Staff encouragement of exercise was similarly attributed to the film. For example, a nephrologist stated that as a result of seeing the film, both she and other health care providers on the unit “are talking more with the patients about exercise. We are trying to encourage [patients] to exercise more.” This change in how health care providers were engaging with patients about exercise was also noted by a clinical manager on the unit who, for example, observed a nurse good-naturedly teasing a patient using an exercise bike, “[Y]ou’ll be … [100km from here] by ... ten o’clock!” She added that such encouragement “reinforces the fact that the patients [are] doing the right thing, that it’s the right thing for them to do … I think it also helps other patients that are not involved with the study see that exercise is a good thing.”

### Realism and aesthetics

In *Fit for Dialysis*, the aesthetic qualities of the film were essential to the perceived realism of the film. Realism was expressed by participants in terms of seeing themselves in the characters, which allowed them to identify with what was being performed, to suspend disbelief about the possibility of exercising while being dialysed, and to imagine what was depicted as a possibility for their own lives. Key to such identification was the linear structure of dramatic action, or what is typically referred to as the “story line” of the film [49]. For example, patients recounted how they identified with Marvin, the main protagonist, particularly with the story line that culminated in his transformation from struggling to pick up a newspaper to bounding down the stairs to retrieve his newspaper with ease. Even our female patient participants identified with the character of Marvin, suggesting that this character identification may not be gender-specific. As the following female patient described, Marvin’s transformation instilled hope that she too could achieve this change:

[At] the beginning [of the film] when [Marvin] was really lethargic, I connected with that right away being lethargic. At the beginning when he barely made it down the steps and he didn’t want to go anywhere with his wife and he didn’t want to do anything. That I related to right away…[and] the energy level that [he] showed towards the end… it gives you the idea that you can have a little more energy…the ending was the one part that got me. It’s just that it’s something you can achieve, possible [to] achieve…it just made me feel good to think maybe I have a chance, you know? Maybe I’ll feel better.

The degree to which character identification was made also largely hinged on the actor’s ability to embody an older, ill, and fatigued patient undergoing hemodialysis. For example, many participants reflected on Marvin’s movements, facial expressions, and gestures. The following comments are particularly noteworthy:

[T]hat first scene when [Marvin] is getting out of the house to pick up the paper, and you can see how much he struggles with every step, he struggled. The movement is a struggle, his whole body language shows you that he is just very, very tired and unfit and you know, then he engages in exercise, you definitely see the change in his whole physique and his body language and his changing performance, the way he walks is different at the end. And I think that the physical aspect of that is one aspect, but also the - you can see the variety of emotions - I think initially a feeling of hopelessness and complete lack of energy and stamina. (Nephrologist).

Well, at the beginning [Marvin] looked old … and like he had a lot of pain and no hope. There was no hope in his eyes. And afterwards, at the end, it looked like he’d straightened up, the look on his face wasn’t as tight as it was at the beginning, he was more relaxed looking and he had a smile on his face. And that’s what I remember of the film mostly is [Marvin’s] face at the beginning and at the end. (Patient)

These same scenes were frequently remarked on by family carers who appeared acutely aware of Claire’s responses to Marvin as his carer. This suggests that among different viewers the same scenes can powerfully evoke identification with different characters based on the participants’ real world location:

I think just seeing his, like the wife too, like having the wife in the scene and seeing her, you know, fretting over him trying to get down the stairs and pick [the newspaper] up and then she’d go and do it for him. Like that was, truly, like, difficult to watch … I know they’re actors but still, you know, you think, well actually that’s terrible, that sucks! And then to see her at the end watching him and he’s, you know, bounding down the stairs and he wants to go to the movies or wherever they went, it was great! (Family carer).

Another example of how the aesthetic qualities of the film supported participants’ identification was the strong resonance reported by health care providers between the dialogue in the film and their own clinical experiences. For example, the following quote from a nephrologist describes her reflection about the scene in which two nurses express exasperation about the possibility of integrating exercise into current hemodialysis care given their workload stresses (Scene 4). In particular she describes the dialogue as both realistic and engaging:

I think [one of the nurses in the film] mentioned … that, you know, we don’t have time to [encourage exercise]…so that was also I think really well-portrayed, taken from real life situations. I can absolutely imagine a discussion like that in our unit with responses like that.

This same nephrologist also reflected on the nephrologist depicted in the film. Specifically, this nephrologist identified with the dismissiveness of the nephrologist in the film about introducing exercise for wellness in the nephrology program:

You know, it was sort of an eye-opener because I am a physician, and in a sense, you know, what I saw in that movie was a physician who is running around without really time commitment [sic] to [discuss patient participation in exercise with] the patient and physiotherapist, and you know, I think in day-to-day practice many times we sort of do it in real life experience. So I thought that, you know, it was depicted in that film very well … it was something that caught my attention to stop and re-evaluate, you know, my position in real life.

In some cases, the resonance of the dialogue and the embodied performance were so strong that it caused discomfort. For example, a clinical manager described her discomfort with her identification with the ‘closed-mindedness’ of the nurses in Scene 4:

Well, for me, I guess … you know, someone that is a registered nurse and has been a hemodialysis nurse, I could see where she was coming from. But I could also see that, you know, perhaps I, you know, I wouldn’t want to identify with her either because I wouldn’t want to be so closed-minded about something that is clearly, you know, has been shown by research to have benefit. So I think it made me feel uncomfortable, to be quite honest, as a registered nurse … I think it was just – I think it was honest … but you know, maybe that’s what I’m feeling the discomfort about; I think it was honest, I think that that is the reaction of many nurses.

A nephrologist expressed similar discomfort with this scene, particularly with the prospect that nursing staff in her practice have similar perceptions regarding patient exercise:

I think the [scene] that had the biggest impact was when the patient comes to the dialysis unit and the nurse, and he’s [asking] about … exercising … and the nurse saying, “I don’t think that’s a good idea, you know, you shouldn’t do that.” So that, you know, the negative interaction I think had a really big effect on me. It felt really bad, really negative. And it made you stop and think, you know, is that really what other people’s perceptions are, like would our [nursing] staff have the same feeling or is it just a dramatization. But then I sort of wondered maybe people do have those thoughts, which is a real shame actually. Because you saw that, you know, it was very negative for the patient … you could get the sense that the patient obviously felt quite discouraged at that point.

Of all the participants, only one (a patient) perceived the film negatively, citing the aesthetics as the reason. As he stated “it’s not going to win an Oscar … It was like one of those infomercials on T.V.” Despite being an older man himself, he also did not like the choice of making the lead characters “the old guy and the old lady” noting that “kidney disease … affects young as well as old” and “it’s not an old person’s disease.” His inability to identify with the lead characters is strikingly different from the other participants’ experiences, which suggest powerful identification with the characters and with what was being performed.

## Discussion

Older adults living with ESRD are at increased risk for frailty, mobility disability, and cardiovascular mortality [1, 2]. Thus, practice guidelines for older adults with chronic kidney disease, including those living with ESRD, recommend regular exercise for wellness [12, 13] Yet a gap persists between best practice guidelines and nephrology care [14–16]. Research suggests the need for a shift in the culture of hemodialysis care towards making exercise prescription, counseling, and assessment a standard part of care [14, 15, 17]. Education for health care providers, patients, and family carers is a key strategy for enabling such a shift. While there has been a recent upsurge of interest in the use of film for education, there is a paucity of evaluation research on this novel knowledge translation strategy, and it has yet to be applied in the context of hemodialysis to support exercise for wellness for older patients.

Our findings suggest that engagement with *Fit for Dialysis* by health care providers, patients, and family carers increased their awareness of the benefits of exercise for older hemodialysis patients, and ameliorated fears of adverse outcomes. Further, motivation for patients to engage in exercise, and motivation of family carers and health care providers to encourage patient exercise was attributed by participants to their engagement with *Fit for Dialysis*. These findings are significant when we consider that research on the factors that enable and constrain exercise in hemodialysis care has identified concerns of patients, health care providers, and family carers over patient safety (e.g. fears of falling, damaging the fistula or chest line, drop in blood pressure), and lack of support for patient exercise by friends, family carers, and health care providers [15, 18].

There is increasing evidence that research-based drama is an effective pedagogical tool and is well-received by health care providers across disciplines (e.g. medicine, nursing, allied health) and training levels [50–53]. Research-based dramatic performance has been found to be particularly effective in fostering emotional engagement because it privileges the complexity of everyday life. Drama as an aesthetic form also evokes experience rather than presents it, which gives research-based drama a uniquely compelling emotional quality [54, 55] that makes it difficult to avoid or intellectualize the struggles portrayed [56].

Drama, including film, has its roots in oral traditions of storytelling, offering a more persuasive narrative structure for the communication of a health message [20, 21]. Further, film is an entertaining medium that engages multiple modalities to foster a greater “sense of reality” that may enhance audiences’ willingness to attend to information conveyed [22, 57, 58]. However, changing practice requires more than ‘a good story.’ Research suggests that audiences such as health care providers, who are oriented toward empiricism, appear to be more receptive to educational dramas when they know that they are based on empirical research [54, 59]. Thus, while health care providers in our study did not explicitly attribute the believability of the dialogue to the research base of *Fit for Dialysis,* here we extend the insights of prior research [54, 59] to argue that perceived sense of relevance and identification is supported by the research base of the dramatization. Our findings suggest that the same can be said of patients’ and family carers’ perceived sense of relevance and identification. Such relevance and identification can also be attributed to CRARUM, the theoretical knowledge translation framework that informed the development of the film. Indeed, a basic premise of the framework is that when tailoring the key messages of the knowldege translation intiative to fit the targeted setting and audience, specific attention is needed to “the (local) mix of conditions and events” [26] (e.g. key stakeholders, types of interactions and activities) to maximize relevance, feasibility, and impact.

While the research-base of the drama is important for fostering participants’ sense of relevance and identification, the mere linear translation of research findings into dramatic form is not sufficient to achieve this. Indeed dramatic reinterpretation (e.g. producing composite characters, emphasizing multiple tellings of the same event, music) has been identified as key to moving audiences to critical awareness about taken-for-granted understandings, assumptions, and practices, and to triggering practice change [26, 35, 60, 61]. This is consistent with our findings that demonstrate that the aesthetic qualities of *Fit for Dialysis* – the story line, the acting, and dialogue – were the generative mechanism for participants’ identification, critical awareness, and envisioning of new possibilities for hemodialysis care. CRARUM explicitly advocates the use of the arts for knowledge translaation in order to facilitate critical reflection and engagement; aesthetics are key to facilitating tangible change [26].

The story line of a film typically has three parts (e.g. the set-up, confrontation, resolution), and introduces a character (or several) who is involved in a series of related incidents or conflicts that lead to a confrontation, followed by a resolution [49]. The unfolding story line of *Fit for Dialysis* moves from a place of fatigue and reduced physical capacity, to energy and action. This is conveyed with Marvin’s transformation from struggling to pick up a newspaper, to engaging in exercise and moving more freely. This transformation was achieved only because of his confrontation with hemodialysis staff who were resistant at first to incorporating exercise into his care. Even his change in costume, from long-sleeved dark clothing to brighter short-sleeved clothing, visually conveys this story line. The unfolding story line is thus “the center … [or] the dynamic force” [49] that sets up the action and moves it forward in a meaningful way “thereby eliciting suitable responses from a concerned audience” [62]. A good story line enables audiences to identify with the characters, and care about the outcome of their story, something that has been shown to enhance the effects of visual initiatives on knowledge/attitudes and practices [63, 64].

Other aesthetic qualities that were critical to character identification were the actors’ skill and the dialogue. Participants commented on Marvin’s dispositions, movements and gestures, and specifically his change from being hunched-over and lethargic to being upright and energetic as a result of participation in exercise. They further noted the strong resonance of particular scenes, interactions, and dialogue with their own experiences. As Saldaña has argued, an effective research-based drama must have both “artistic rigor” and a “truthful text” – what he refers to as “ethnodramatic validity” [65]. Our exploration of why *Fit for Dialysis* was effective affirms this, but also importantly contributes to the evidence base of the use of film for health education in demonstrating the importance of aesthetics for the perceived sense of relevance and identification. In particular, while previous quantitative research on educational film has found that identification may be related to a perceived similarity between characters and audiences, and perceived realism (e.g. typicality, factuality) [22, 63, 66], ours is the first study to show that aesthetic qualities of a research-based film also support identification.

### Study strengths and limitations

Our findings suggest that identification with the lead character Marvin may not be gender specific, yet our small number of female patients did not allow us to explore this in sufficient depth. While only one patient in our study, himself an older man, expressed an inability to identify with Marvin, this does suggest that the issue of identification is complex and requires further research to identify the key ingredients for optimizing resonance of other research-based films. Despite these limitations, we did find that the aesthetic qualities of *Fit for Dialysis* were critical for its perceived effectiveness, which is reflected in all but one of the participants’ responses. This is significant given that prior research suggests that exploring the aesthetic qualities is necessary to better understand the particularities of drama that work so well as a knowledge translation strategy [35, 61, 67]. Yet, much more work is needed to understand how the aesthetic qualities perceived by our participants (e.g. character, story line, dialogue), and still other such qualities (e.g. costume, props, music) can inform the development of effective films for health education.

## Conclusions

There has been a call to shift hemodialysis care from providing medical treatment alone to a wellness-focused approach that incorporates exercise counselling and support, and participation as part of routine care [14, 15, 17]. However, there remains a paucity of innovative approaches to developing educational initiatives despite that innovation is key to affecting such a shift. Research-based film offers precisely the innovation needed to bring about the transformational shifts required to achieve a culture of exercise for wellness in hemodialysis care. *Fit for Dialysis* is thus well-positioned to optimize the health and wellbeing of older adults living with ESRD.

## Abbreviations

CRARUM: Critical Realism and the Arts Research Utilization Model
ESRD: End-stage renal disease
